# Matrine disturbs the eimeria necatrix-induced loop of tuft cell-intestinal stem cell-goblet cell by inactivating IL-13/JAK2/STAT3 signaling

**DOI:** 10.1016/j.psj.2025.104786

**Published:** 2025-01-10

**Authors:** Geng-xiu Zan, Hao-zhan Qu, Jia Meng, Xiao-fan Wang, Hui-chao Yan, Xiu-qi Wang, Jia-yi Zhou

**Affiliations:** State Key Laboratory of Swine and Poultry Breeding Industry/College of Animal Science, South China Agricultural University/Guangdong Laboratory for Lingnan Modern Agriculture/Guangdong Provincial Key Laboratory of Animal Nutrition Control, Guangzhou, 510642, China

**Keywords:** Chick, *Eimeria necatrix*, Matrine, Tuft cell-intestinal stem cell-goblet loop, IL-13/JAK2/STAT3 signaling

## Abstract

As sensors in the gut, tuft cells integrate a complex array of luminal signals to regulate the differentiation fate of intestinal stem cells (ISCs), which trigger a loop of tuft cell-ISC-goblet cell after parasitic infection. As a plant-derived alkaloid, Matrine plays a prominent role for standardizing ISC functions in *Eimeria necatrix* (EN)-exposed chicks. In this study, we investigated the modulation effects of Matrine on the specific intestinal epithelial cell loop in EN-exposed chicks *in vivo* and intestinal organoids (IOs) *ex vivo*. The results showed that EN infection resulted in swelling and hemorrhage of the jejunum, accompanied by the increase in levels of sIgA and inflammatory cytokines (IL-6, IL-1β, and TNF-α). And these inflammatory symptoms were effectively relieved by Matrine intervention. Concurrently, Matrine resisted the EN-induced increase in tuft cell numbers and levels of crucial pro-inflammatory factors (IL-25 and IL-13), while also reversing the differentiation of secretory cell progenitors into goblet cells. Importantly, Matrine impeded the upregulation of the inflammatory signaling pathway JAK2/STAT3 in EN-infected chicks and IOs. Conversely, exogenous supplementation of IL-13 or activation of STAT3 via Colivelin eliminated the standardization of the tuft cell-ISC-goblet cell loop by Matrine. Overall, our findings suggested that Matrine intercepted the tuft cell-ISC-goblet cell loop by reinstating IL-13/JAK2/STAT3 signaling after EN infection.

## Introduction

Chicken is the good source high-quality and cost-effective protein for the growing human population (Abbas et al., 2019; Abbas, 2020). However, a range of parasites have serious negative effect on poultry health and production performance (Abbas et al., 2016). Among them, Avian coccidiosis is predominantly prevalent in south-central Asia (China, India), the Middle East (Iran, Pakistan), northern Africa (Nigeria, Ethiopia), and certain parts of the Americas (United States, Colombia), causing an economic loss of approximately $2-3 billion annually (Badri et al., 2024; Györke et al., 2013). To date, researchers have identified 7 species of coccidids in chicken, namely *Eimeria tenella* which invades the cecum, *Eimeria brunetti* that causes damage to the terminal part of the digestive tract, and *Eimeria necatrix, Eimeria maxima, Eimeria acervulina, Eimeria mitis and Eimeria praecox* that parasitize the small intestine. In comparison, *Eimeria necatrix* (EN) inflicts more severe damage on the host's intestinal tract (Fernando et al., 1983; Mattiello et al., 2000). Upon ingestion by chicks, the oocyst of EN release sporozoites in the stomach due to digestive enzyme activity. Subsequently, these sporozoites enter the intestinal lumen along with chyme and utilize their trypanosome-like and rod-like structures to adhere to and invade epithelial cells (Ruiz et al., 2015; Soldati et al., 2001). Finally, the parasite undergoes rapid proliferation to produce new schizonts, gametocytes, and oocysts by exploiting intracellular nutrients, which cause intestinal structural damage and impair nutrient absorption (Wang et al., 2021b). Moreover, EN infection also induces intestinal inflammation, leading to tissue lesions and an increased susceptibility to other microbial infections, thereby posing a serious threat to host intestinal health (Constantinoiu et al., 2011).

The diverse functional intestinal cells, derived from intestinal stem cells (ISCs), are fundamental to the biological activities of the intestinal epithelium (Clevers et al., 2014). For example, absorptive cells facilitate nutrient transport; goblet cells prevent pathogen adhesion through mucin secretion; Paneth cells produce signaling factors to maintain the ISCs niche; endocrine cells regulate the digestive and absorptive functions by releasing gastrointestinal peptides; and tuft cells perform immunosurveillance and transmit immune signals (Beumer and Clevers, 2021). The fate of these differentiated cells is not only co-regulated by multiple niche cells, among which lamina propria immune cells are an important source of signaling (Corrêa et al., 2023; Sato et al., 2009).

When the intestinal immune system is activated, signal factors produced by immune cells directly intervene in the ISC commitment lines (Biton et al., 2018). Researchers have discovered that tuft cells within the intestinal epithelium are responsible for monitoring worm infestation and promptly producing interleukin 25 (IL-25) to activate type 2 innate lymphoid cells (LC2), which in turn secrete IL-13 to induce ISC differentiation into tuft cells and goblet cells (von Moltke et al., 2016). Interestingly, the process of EN invasion is accompanied by a significant accumulation of inflammatory factors and a propensity of ISCs towards the secretory cell fate in intestinal tissues (El-Ghareeb et al., 2023; Salas et al., 2020; Zan et al., 2024). However, it remains unclear whether EN promotes a sustained increase in goblet cells through a loop of tuft cell-ISC mediated by inflammatory pathways.

Prolonged utilization of traditional ionophoric antibiotic drugs may lead to significant multidrug and cross-drug resistance in coccidids. Consequently, the development of green and efficient anticoccidial alternatives has become an urgent priority for the poultry industry (Hayajneh et al., 2024). Plant extracts, which contain a variety of natural active compounds such as polysaccharides, polyphenols, alkaloids, saponins, and alcohols, exhibit multiple bactericidal effects and play crucial roles in immune regulation, antioxidation, and anti-inflammation (Ahmad et al., 2023). This multifunctionality makes it challenging for pathogens to develop resistance to these compounds. For instance, star anise extracts and citrus sinensis essential oil have demonstrated excellent anti-coccidian efficacy (Salman et al., 2022; Al-Hoshani et al., 2023). Hailat et al. (2024) compared the efficacy of plant extracts with chemical anticoccidials (nicarbazin and narasin) and found that the chemical drugs did not significantly outperform the plant extracts in restoring growth performance in coccidia-infected chickens. Matrine is a traditional Chinese medicine ingredient whose ability to kill protozoa and alleviate inflammatory response has been confirmed by many studies (Ali et al., 2017; Li et al., 2019). We have previously reported that Matrine could effectively mitigate EN-induced ISC injury of chicks (Zan et al., 2024). In this study, we further revealed that Matrine blocks the EN-induced tuft cell-ISC-goblet cell loop by reinstating IL-13/JAK2/STAT3 signaling, which enriches the theoretical foundation of Matrine as a protective agent against EN injury.

## Materials and methods

### Oocyst isolation and purification

The intestinal contents of EN-infected chicks was collected, subsequently mixed with ddH_2_O and filtered using a mesh screen to remove impurities. The resultant mixture was centrifuged, suspended in saturated salt water, centrifuged once more, and the supernatant was collected. Finally, the supernatant was diluted by adding 9 times of ddH_2_O, and the precipitation obtained through centrifugation was the oocyst (stored at 2-8°C).

### Animal experiment

A total of 96 1-day-old yellow-feathered broilers were divided into 4 groups with 8 replicates per group and 3 chicks per replicate. The control (CON) and EN group were fed a basal diet, Matrine and EN + Matrine groups (EN+M) were fed a basal diet supplemented with 350 g/t Matrine (#M813524, 98% purity, MACKLIN, Shanghai, China). And chicks in the EN group and the EN+M group was received a single gavage of 15,000 oocysts on the 15th day, then were euthanized after 7 days. All experiments were approved by the animal ethics committee of South China Agricultural University (SCAU#0241, Guangzhou, China).

### *Ex vivo* assay

First, the intestinal organoids (IOs) were exposed to 0, 1, 10, and 100 ng/mL IL-13 protein (Human, HY-P70568, MedChemExpress, NJ, USA) or 0, 0.1, 0.5, and 2.5 μM Colivelin (#S9664, Sellect, TX, USA) to determine the optimal treatment concentration based on IO growth advantages. Subsequently, the IOs were divided into 8 groups: CON (treated with PBS), Matrine (treated with 20 μg/mL Matrine), EN (treated with 15,000 oocysts), IL-13 (treated with 100 ng/mL IL-13), Colivelin (treated with 0.5 μM Colivelin), EN + Matrine (treated with 15,000 oocysts and 20 μg/mL Matrine), EN + Matrine + IL-13/Colivelin (treated with 15,000 oocysts, 20 μg/mL Matrine, and 100 ng/mL IL-13/0.5 μM Colivelin) group. The growth of IOs was monitored over a period of 4 consecutive days.

### Antibodies

Antibodies against Atoh1 (#A6530, ABclonal, Wuhan, China), DCLK1 (#ab31704, Abcam, Cambridge, UK), IL-1β (#A16288, ABclonal), IL-6 (#ab233706, Abcam), p-JAK2 (#381556, ZENBIO, Chengdu, China), p-STAT3 (#AP0705, ABclonal), SOX9 (#380995, ZENBIO), Tumor Necrosis Factor α (TNF-α, #ab183218, Abcam) and β-actin (#380624, ZENBIO, Chengdu, China), as well as anti-rabbit IgG (#511203, ZENBIO) and anti-mouse IgG (#511103, ZENBIO) antibodies, were used for Western blotting or Immunohistochemistry staining.

### Intestinal lesion scoring criteria

The intestinal tissues of chicks in the CON, Matrine, EN, and EN + M groups were dissected longitudinally, and the intestinal lesion score was assessed in accordance with Table 1.

**Table 1:** Intestinal lesion scoring criteria.

Score	Scoring criteria
0	The intestinal wall has no visible lesions
1	The intestinal wall is not thickened and there is minor bruising, and the content is normal
2	The intestinal wall appears markedly lesioned and thickened, and the content is visibly hemorrhagic
3	The intestinal wall marked thickening, more bleeding in the lumen, presence of blood clots or banana-like lumps
4	Intestine filled with large amounts of blood clots swelled intestinal wall, or death of the chick

### Paracellular permeability assay

Fresh jejunal tissues of chicks were collected and carefully stripped of the myoplasmic layer, and trans-epithelial electrical resistance (TEER) values were obtained by Ussing Chamber according to the previous study (Zan et al., 2023).

### Elisa kit detection

The concentrations of sIgA (#ml002778, Mlbio, Shanghai, China), IgG (#ml092483 Mlbio), IL-13 (#ml023399, Mlbio), and IL-25 (#YJ625024, Mlbio) in the serum or tissue were detected by Elisa kits according to the instructions.

### RNA extraction

The frozen intestinal tissue was ground into a powder, added to trizol, and extracted using chloroform. Subsequently, the RNA was precipitated and washed with 75% ethanol. Finally, cDNA was obtained using a reverse transcription kit. The RNA of IOs was obtained using SuperPrep cell Lysis & RT Kit (#SCQ-101, Toyobo, Osaka, Japan).

### Real-Time qPCR

After the target gene primer was designed, the mRNA abundance was detected following the previous study (Zhou et al., 2020) and calculated by 2^−ΔΔCt^ method. The primers for the target gene are presented in the supplementary materials (Supplementary Table 1).

### Preparation of paraffin sections

The fresh intestinal tissue was fixed in 4% paraformaldehyde for 24 hours and then processed by dehydration and immersed in xylene for 30 min. Finally, the sample was embedded in paraffin wax and cut into 5-μm slices by a microtome.

### Periodic Acid-Schiff (PAS) stain

The paraffin section was stained by a PAS staining kit (#C0142S, Beyotime, Shanghai, China).

### Immunohistochemistry

The paraffin-embedded section was deparaffinized in xylene. After rehydration and antigen repair treatment, the sample was sequentially blocked with BSA, incubated with primary antibody and secondary antibody. The nuclei were counterstained by 4′,6-di-amidino-2-phenylindole (DAPI). Finally, fluorescence signals were captured using inverted fluorescence microscopy (NIS-Elements, Nikon, Japan) and analyzed using ImageJ software (version 1.8.0 112, National Institutes of Health, Bethesda, MD, USA).

### Western blotting

First, protein samples and markers (M221-10, GenStar, Beijing, China) were loaded onto the SDS gel for electrophoresis. Subsequently, they were transferred to polyvinylidene fluoride (PVDF) membranes, blocked with sealing fluid (New Cell & Molecular Biotech Co., Ltd., Suzhou, China), and incubated with primary and secondary antibodies. Finally, the membrane was mixed with luminescent solution (#MK-S500, MIKX, Shenzhen, China) to obtain data using Fluor Chem M imaging system (Protein Simple, San Jose, CA, USA).

### Data analysis

The data were analyzed using SPSS software version 19.0 (SPSS Inc., Chicago). Differences among four or six groups following a standard one-way ANOVA were evaluated using the least significant difference (LSD) multiple-range tests. The data were expressed as the mean ± SEM, with significance levels denoted by *p < 0.05, ^⁎⁎^*p* < 0.01, and ^⁎⁎⁎^*p* < 0.001, while statistical tendencies were indicated by 0.05 ≤ *p*-values < 0.10.

## Results

### Matrine alleviates EN-induced jejunal inflammation

**Fig. 1:**
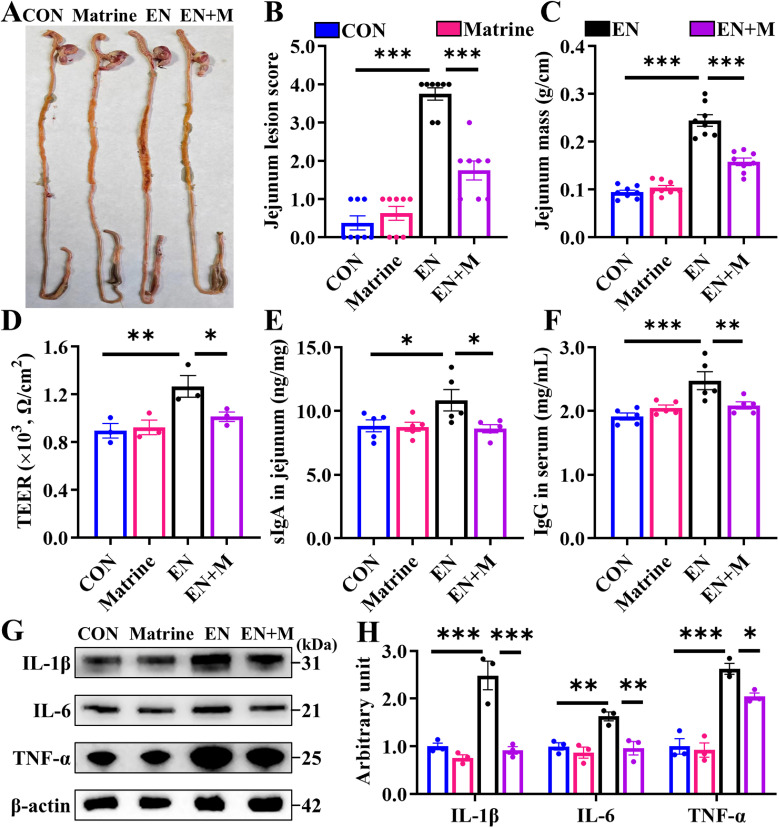
Matrine improves the jejunal inflammation in EN-infected chicks. A total of 96 chicks were divided into CON, Matrine, EN and EN+M groups and fed the basal diet and the diet supplemented with 350g/t Matrine, respectively. On the 15th day, the chick in the EN group and the EN+M group was given a single gavage of 15,000 oocysts and the trial ended 7 days after infection. (A) The image of the gastrointestinal tract; (B) Jejunum lesion score (*n* = 8); (C) Jejunum mass (*n* = 8); (D) TEER of the jejunum (*n* = 3); (E) The level of sIgA in jejunum (*n* = 5); (F) The level of IgG in Serum (*n* = 5); (G-H) The expression of IL-1β, IL-6, and TNF-α in the jejunum was detected by Western blotting (*n* = 3). The results are expressed as the mean ± SEM. *p < 0.05, **p < 0.01, ***p < 0.001.

In the *in vivo* experiment, we observed hemorrhages and swelling in the jejunum of chicks in the EN group with severe lesions (Fig. 1A-B). The data indicated a significant increase in jejunal mass (Fig. 1C) and TEER value (Fig. 1D) after EN infection. In addition, compared with the CON group, levels of IgG, sIgA, and the pro-inflammatory cytokines (IL-1β, IL-6, and TNF-α) were significantly elevated in the serum or jejunum (Fig. 1E-H). However, these inflammation indicators were reversed after Matrine supplementation (Fig. 1A-H). These results suggest that Matrine exhibits anti-inflammatory properties and restores immune homeostasis within the jejunum of EN-infected chicks.

### Matrine blocks the EN-induced Loop of tuft cells-ISCs-goblet cells

**Fig. 2:**
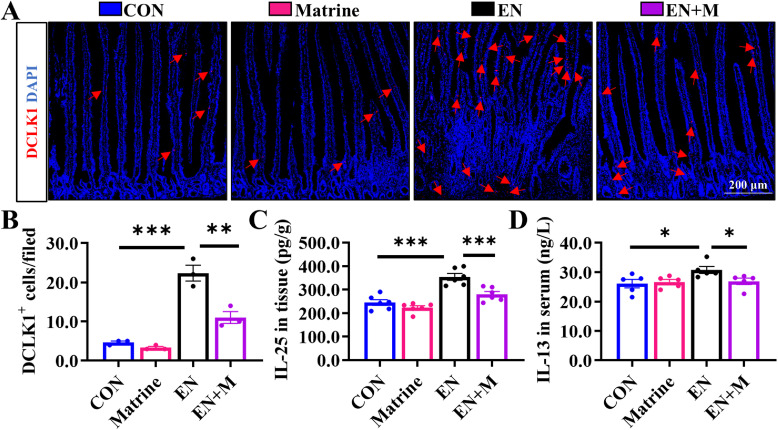
Matrine prevents differentiation of chicken ISCs to tuft cells after EN infection. (A-B) IHC staining of DCLK1 (the marker of tuft cells) proteins in the jejunum (100×) (*n* = 3); (C) The level of IL-25 in jejunum (*n* = 6); (D) The level of IL-13 in serum (*n* = 5); The results are expressed as the mean ± SEM. *p < 0.05, **p < 0.01, ***p < 0.001.

**Fig. 3:**
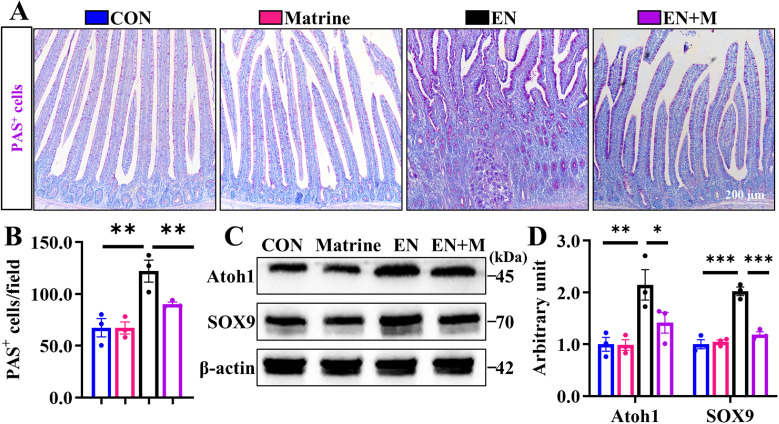
Matrine hinders the EN-exposed ISCs transforming into goblet cell. (A-B) The PAS staining (labeled goblet cells) in jejunum (100×) (*n* = 3); (C-D) Expression assay of Atoh1 and SOX9 (the marker of Secretory progenitor cells) in the jejunum detected by Western blotting. The results are expressed as the mean ± SEM (*n* = 3 animals). *p < 0.05, **p < 0.01, ***p < 0.001.

The stable differentiation lineage of ISCs is a prerequisite for maintaining intestinal health. EN increased the number of DCLK1-labeled tuft cells (Fig. 2A-B) and PAS-labeled goblet cells (Fig. 3A-B) in the jejunum. Furthermore, EN-infected chicks exhibited elevated levels of IL-25 and IL-13, crucial pro-inflammatory factors involved in tuft cell differentiation (Fig. 2C-D), as well as enhanced expression of secretory progenitor cell marker proteins Atoh1 and SOX9 (Fig. 3C-D). Importantly, these indicators returned to the CON group level upon treatment with Matrine group, which suggested that Matrine blocks the EN-induced loop of tuft cell-ISC-goblet cell.

### Matrine inhibits JAK2/STAT3 signaling pathway in EN-infected jejunum

**Fig. 4:**
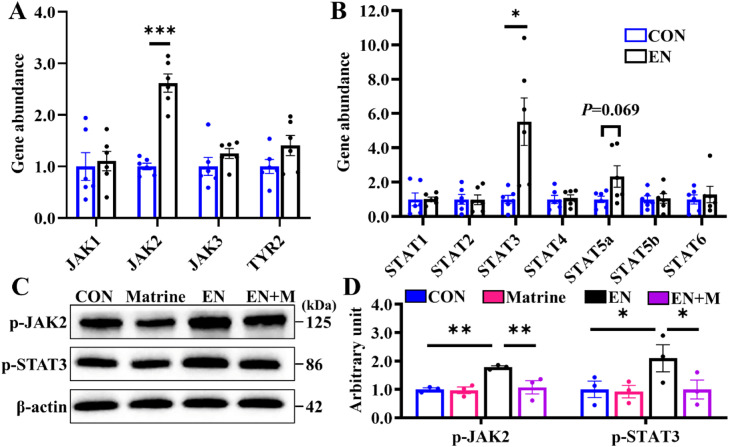
Marine blocks the hyperactive of jejunal JAK2/STAT3 signaling after EN infection. (A-B) The gene abundance of *JAK* family members (*JAK1, JAK2, JAK3, TYR2*) and STATs family members (*STAT1, STAT2, STAT3, STAT4, STAT5a, STAT5b, STAT6*) in CON and EN group; (C-D) The expression of p-JAK2 and p-STAT3 in the jejunum detected by Western blotting. The results are expressed as the mean ± SEM (*n* = 3 animals). *p < 0.05, **p < 0.01, ***p < 0.001.

JAK/STAT signaling serves as a crucial pathway for multiple interleukins. Our data revealed a 1.6-fold increase in *JAK2* mRNA abundance in the jejunum of the EN group compared to the CON group, while no significant differences were observed in the abundance of *JAK1, JAK3*, and *TYR2* (Fig. 4A). Meanwhile, among the STAT family members, only *STAT3* and *STAT5a* gene levels exhibited a 4.5-fold and 1.3-fold increase respectively following EN exposure in the jejunum (Fig. 4B). Furthermore, the Western blotting analysis confirmed that EN upregulated p-JAK2 and p-STAT3 expression levels; however, Matrine prevented the cascade amplification of JAK2/STAT3 signaling (Fig. 4C-D).

### IL-13/JAK2/STAT3 signaling functions as an important hub for Matrine-initiated repair after EN injury *ex vivo*

**Fig. 5:**
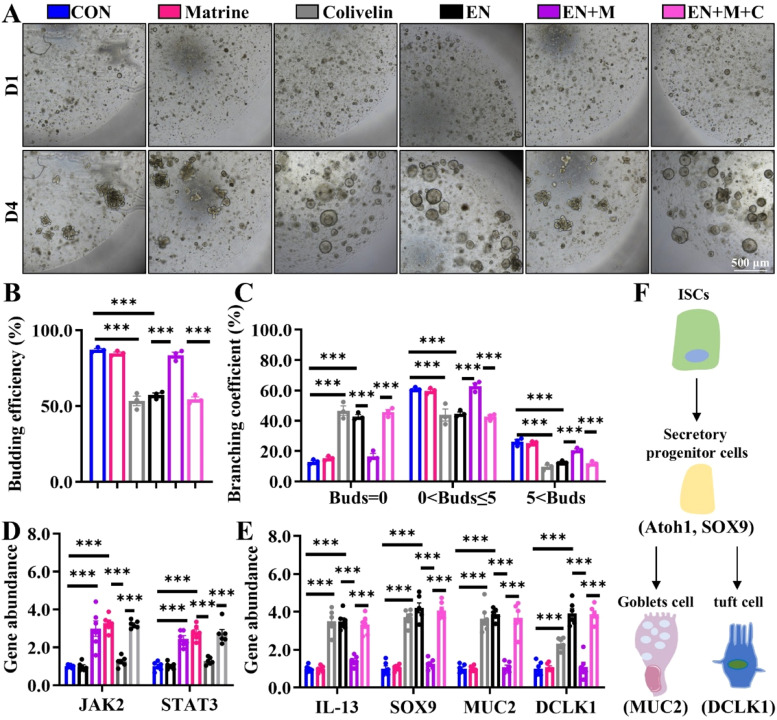
Supplementation with IL13 eliminates the beneficial effects of Matrine on the IOs of chicks under EN invasion. The IO was treated *in vitro* with PBS, Matrine (20 μg/mL), EN (15000 oocysts), IL-13(10 ng/mL), EN+Matrine, IL-13+ EN+Matrine (two replicates per group, each containing two wells of organoid) for 4 days. (A) Representative images of IOs cultured from each group (40×); (B-C) The results of the statistical analysis of organoid bud number and branching coefficient of organoids (*n* = 3); (D-E) The levels of mRNA expression for *JAK2, STAT3, IL-13, SOX9, MUC2* (labeled goblet cells) and *DCLK1* (*n* = 6). (F) The differentiation lineage axis of ISCs-tuft cell-goblet cell. The mean ± SEM represents the obtained outcomes. *p < 0.05, **p < 0.01, ***p < 0.001.

**Fig. 6:**
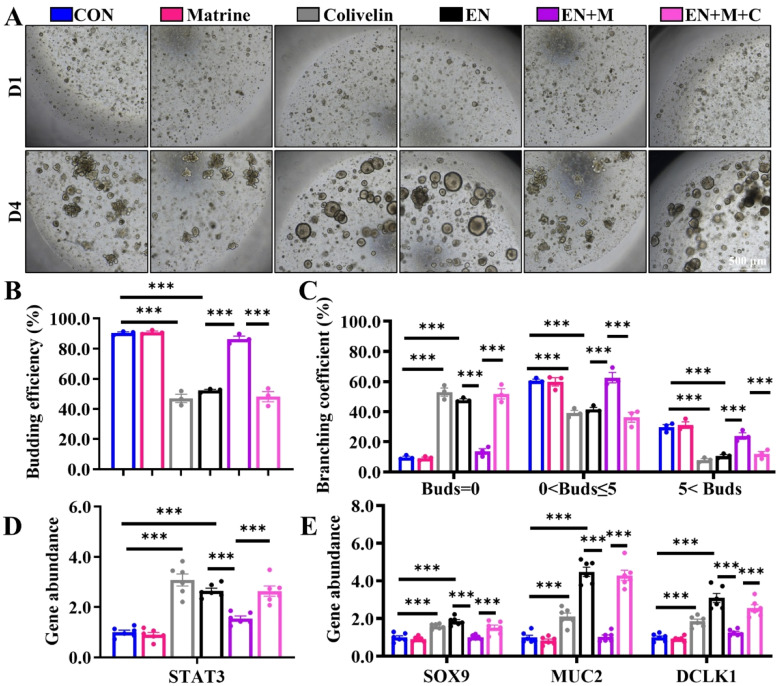
Activating the JAK2/STAT3 signal eliminates the beneficial effects of Matrine on the EN-infected IOs. The effect of Matrine on EN-exposed IOs was evaluated after Colivelin (0.5 μM) activated STAT3 activity in IOs. (A) Representative images of chick IOs (40×); (B-C) The results of the statistical analysis of organoid bud number and branching coefficient of organoids (*n* = 3); (D-E) The expressions of active *STAT3, SOX9, MUC2* and *DCLK1* in IOs (*n* = 6). The results are expressed as the mean ± SEM. *p < 0.05, **p < 0.01, ***p < 0.001.

To investigate the potential of Matrine in mitigating ISC damage by inhibiting EN-triggered IL-13/JAK2/STAT3 signaling, we conducted *ex vivo* experiments using IOs as models. Initially, IOs were treated with varying concentrations of IL-13 or Colivelin (Fig. S1 and S2) and confirmed that 10 ng/mL IL-13 and 0.5 μM Colivelin were sufficient to inhibit the budding efficiency of IOs. The results showed that the proportion of budding IOs in the EN group were significantly reduced (Fig. 5A-C and Fig. 6A-C), and the gene abundance of *JAK2, STAT3, SOX9, MUC2*, and *DCLK1* was significantly increased by EN (Fig. 5D-E and Fig. 6D-E). Matrine exhibited a remarkable ability to enhance the budding efficiency of EN-infected IOs while suppressing JAK2/STAT3 signaling pathway activation and promoting functional cell markers. We also detected that excess Mtrine (40 μg/mL) significantly reduced *Atoh1, SOX9, JAK2* and *STAT3* gene levels (Fig. S3). In contrast, neither IL-13 ligand nor the STAT3 agonist Colivelin could rescue the growth advantage or differentiation fate of ISCs under EN exposure when compared to Matrine. These findings suggest that IL-13/JAK2/STAT3 signaling functions as an important hub for Matrine-initiated repair after EN injury.

## Discussion

The intestinal tract, an organ with the largest contact area with the external environment, is highly susceptible to invasion by external pathogens (Zhou et al., 2024). Upon entry into the digestive tract, harmful substances typically disrupt cellular structure and impair intestinal mucosal function (Bao et al., 2024; Ren et al., 2022; Zhou et al., 2019). In our current investigation, we observed that EN infection caused damage to the jejunum tissue of chicks, resulting in inflammation and hemorrhaging of the intestinal wall. Interestingly, compared with the CON group, both jejunal weight and TEER values exhibited a significant increase following EN infection. This could potentially be attributed to coccidia interfering with homeostatic control centers of ISCs and triggering an inflammatory response (Lang et al., 2009). In our previous study, EN infection stimulated ISC proliferation and enhanced intestinal mucosal thickness in chicks (Zan et al., 2024). Researchers have found that coccidia invasion can initiate a robust immune response within the intestines (Yazdanabadi et al., 2020; Zhou et al., 2023). Consistently, we further confirmed a significant increase in serum IgG, tissue sIgA, and pro-inflammatory cytokines (IL-1β, IL-6, and TNF-α) in EN-infected chicks. In recent years, incorporating plant extracts into livestock and poultry diets has emerged as a significant strategy to enhance animal immune function or mitigate immune stress (Park, et al., 2023). For instance, studies have demonstrated that extracts from Carica papaya, Black Cumin Seed, Artemisia brevifolia, and Citrus sinensis essential oil can effectively bolster the immune response aand reduce pathogen damage (Abbas and Alkheraije, 2023; Hakim, et al., 2019; Imran, et al., 2022; Hussain, et al., 2023). Matrine has also been demonstrated to modulate intestinal immunity and mitigate inflammatory responses during EN exposure. Our previous data indicates that AntiC, a plant extract containing Matrine as the primary active ingredient, safeguards the intestinal health of chicks against EN damage. In addition, as a natural plant molecule, Matrine undergoes efficient metabolism within the body, thereby resolving concerns regarding residual traces of conventional anticoccidial drugs like Monensin and Narasin while significantly enhancing the quality of livestock products (Butaye et al., 2003).

The orderly differentiation of ISCs underlies intestinal epithelial function (Sato et al., 2020). Under various environmental stimuli, ISCs dynamically adjust their differentiation patterns to ensure adaptive responses of the epithelium (Zhou and Boutros, 2023). Cheng et al*.* (2019) demonstrated a high-ketone diet promotes ISC differentiation into absorptive cells, potentially enhancing nutrient absorption. Remarkably, protozoan infection induces an increase in goblet cells and tuft cells, facilitating mucin secretion and preventing worm adhesion (Schneider et al., 2018). Interestingly, EN infection also leads to elevated levels of IL-25 and IL-13. It is highly plausible that EN triggers similar ILC2 signaling to increase IL-13 secretion, thereby amplifying the feedback loops between tuft cell-ISC-goblet cell interactions (Hong et al., 2006). A previous study has demonstrated the effective inhibitory effects of Matrine on IL-13 production and goblet cell reduction in the pulmonary tract (Sun et al., 2016). In the present study, Matrine exhibited remarkable potential in rescuing the differentiation lineage of ISCs infected with EN. However, supplementation with IL-13 nullified the standard ISC fates mediated by Matrine, which confirms the pivotal role of IL-13 as a key mediator for delivering invasion signals from EN to ISCs.

As a crucial bridge of dialogue between interleukins and ISCs, the JAK/STAT signaling was disturbed in the jejunum of EN-infected chicks (Peng et al., 2022; Wang et al., 2021a). Our results indicated that EN exposure significantly upregulated the gene abundance of *JAK2* and *STAT3*. And the inactivation of JAK2/STAT3 signaling hampered the differentiation potential of ISCs. In contrast, STAT3 activation increased goblet cell population within IOs (Herrera and Bach, 2019). In line with these observations, our study revealed that EN stimulated the differentiation of ISCs towards secretory cells by activating JAK2/STAT3 signaling. ISC differentiation is regulated by a collaborative network of multiple signaling pathways, such as JAK/STAT, Wnt, and Notch (Beebe et al., 2010; Jung et al., 2018). In the context of EN injury, the high expression of secretory cell regulatory genes *Atoh1* and *SOX9* may be attributed to the crosstalk within the intracellular signaling network. Notably, Matrine effectively inhibited hyperactive JAK2/STAT3 signaling induced by EN. However, when STAT3 is activated using agonists, Matrine fails to restore the growth advantage of IOs, which provides compelling evidence for the crucial role played by the JAK2/STAT3 signaling pathway in regulating ISC function under EN exposure.

**Fig. 7:**
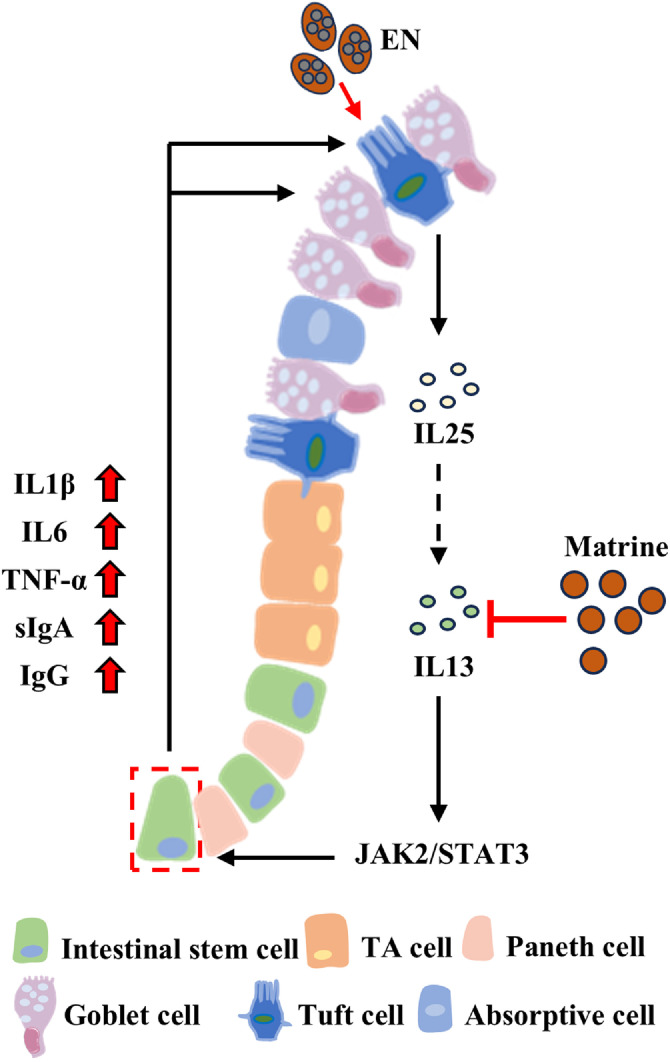
The mechanism of Matrine rescuing ISC fate lineages in EN-infected chicks. Matrine disturbs the Eimeria necatrix-induced loop of tuft cell-intestinal stem cell-goblet cell by inactivating IL-13/JAK2/STAT3 signaling.

Overall, our study highlights that Matrine blocks the EN-induced loop of tuft cell-ISC-goblet cell through IL-13/JAK2/STAT3 signaling pathway, providing new insights into the potential anticoccidial strategy of Matrine (Fig. 7). Intestinal homeostasis is maintained by a complex network of signaling pathways regulating diverse functional intestinal epithelial cells; therefore, further investigation is needed to determine whether Matrine affects other cell types or signaling pathways in response to EN-induced immune responses.

## Author Contributions

G.X.Z. contributed to the investigation, data curation, validation, visualization and writing original draft. H.Z.Q. and J.M. contributed to the data curation and visualizatio. H.C.Y. and X.F.W contributed to the supervision. X.Q.W. and J.Y.Z. contributed to the conceptualization , funding acquisition, methodology and writing review and editing. All authors read and approved the final manuscript.

## Data availability statement

The data that support the findings of this study are available from the corresponding author upon reasonable request.

## Funding Information

This work was supported by the National Key Research and Development Program of China [2024YFD1300805], and the Science and Technology Program of Guangzhou [2024B03J1267].

## Declaration of competing interest

The authors declare no conflicts of interest.

## Appendix Supplementary materials

Image, application 1

## Footnotes

Supplementary material associated with this article can be found, in the online version, at doi:10.1016/j.psj.2025.104786.
