# Supplementary material for: Matrine disturbs the eimeria necatrix-induced loop of tuft cell-intestinal stem cell-goblet cell by inactivating IL-13/JAK2/STAT3 signaling
Source: Poult Sci. 2025 Jan 10;104(2):104786. doi: 10.1016/j.psj.2025.104786 (PMC11954915; doi:10.1016/j.psj.2025.104786)
Supplement: Supplementary file 1 [file mmc1.docx]

**SUPPLEMENTARY INFORMATION**

**
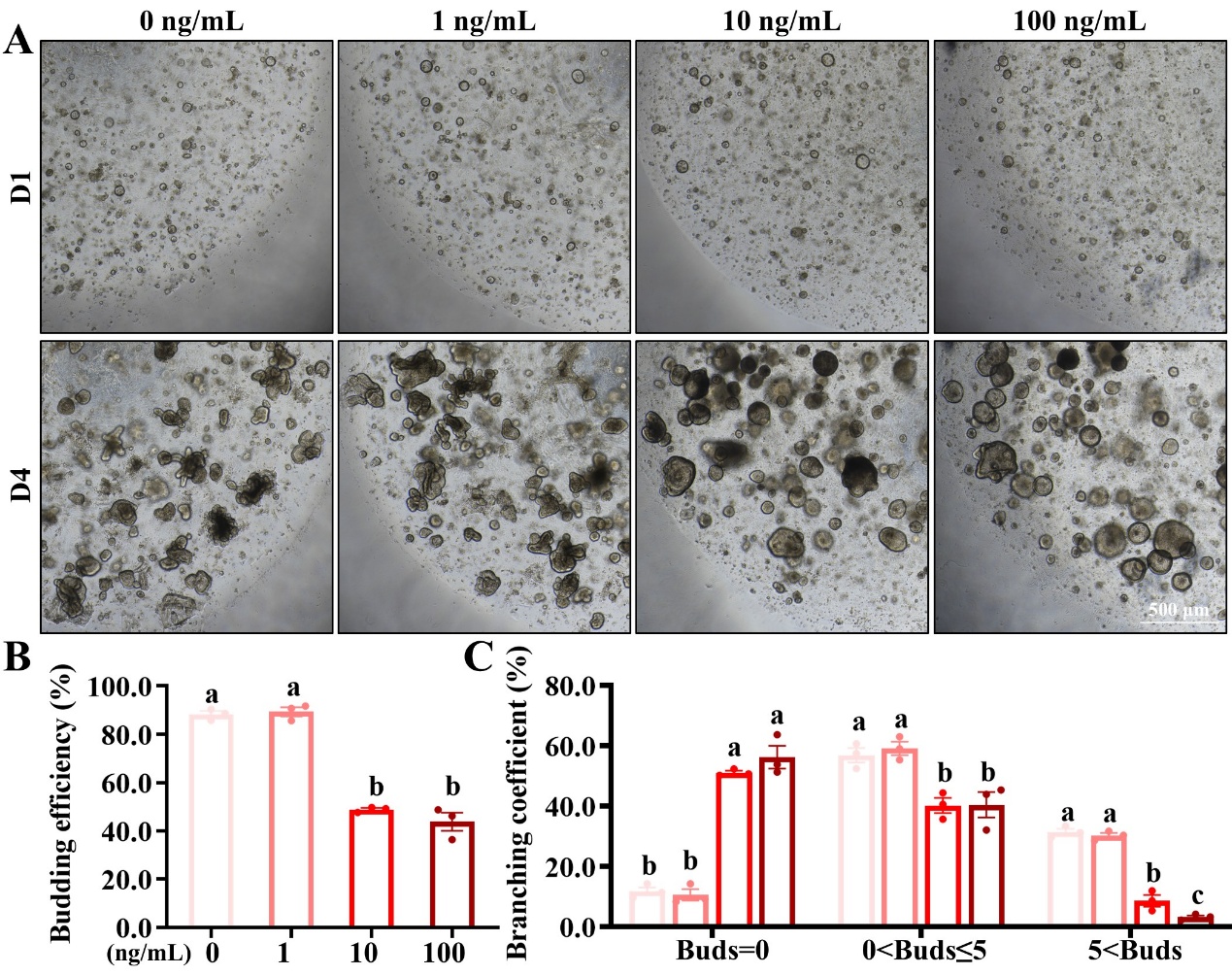
Figure S1 *Ex vivo* dosage screening assay of IL13.**

(A) Representative images of intestinal organoids cultured from chicken crypt cells treated with 1/ 10/ 100 ng/mL IL13;

(B-C) The results of the statistical analysis of organoid bud number and branching coefficient of organoids.

**
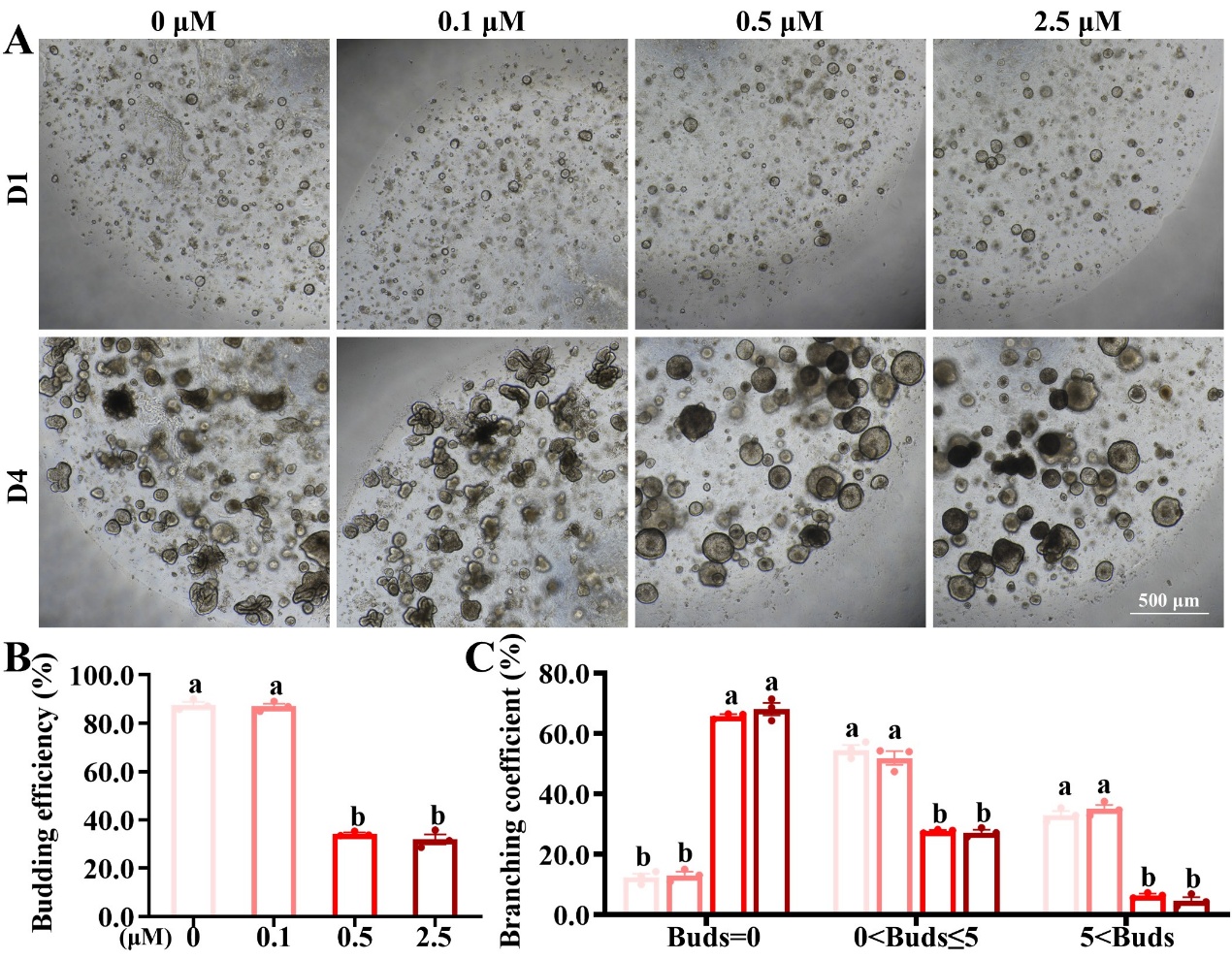
**

**Figure S2** ***Ex vivo* dosage screening assay of Colivelin.**

(A) Representative images of intestinal organoids cultured from chicken crypt cells treated with 0.1/ 0.5/ 2.5 μM Colivelin;

(B-C) The results of the statistical analysis of organoid bud number and branching coefficient of organoids.


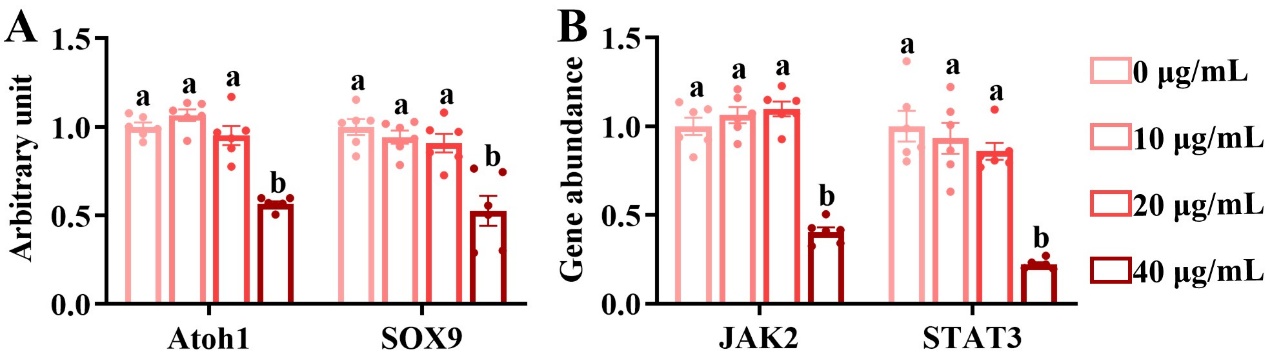


**Figure S3 Effects of different concentrations of Matrine on *Atoh1*, *SOX9*, *JAK2*, and *STAT3* genes in chicken IOs.**

(A-B) The expressions of active *Atoh1*, *SOX9*, *JAK2* and *STAT3* in IOs treated with 10/ 20/ 40 μg/mL Matrine (n = 6).

**Supplementary Table 1 Primers used in this study**

| **Gene** | **Forward primer (5′-3′)** | **Reverse primer (5′-3′)** |
| --- | --- | --- |
| IL13 | ATGACACCAGAGTGGCACAA | GGCAAGAAGTTCCGCAGGTA |
| JAK1 | GCCCGTTGGGGCGAG | GGTAACTCGTGTCTGGCTCTTC |
| JAK2 | GTGCTGAGGGGAAGCCG | GTTCAGAACATTTGCTTGCTGT |
| JAK3 | TCGTAGTGACTTCATCGCGG | CAGGTCTTGTAGCTGACGCA |
| TYR2 | GTGCTGAGGGGAAGCCG | GTTCAGAACATTTGCTTGCTGT |
| STAT1 | GGCTGTTTGTTCCAGCGG | GCTGCGTGTTCCCAATCCT |
| STAT2 | CGTGGAGCCGATGGACAG | AATAAAGGGCTGGGCAACGA |
| STAT3 | CGCACCCTTGACTCACTCAT | AAGGTCAGCGACTCGAACTG |
| STAT4 | AGGGACCACTCGTACAGCAT | GGATGCAGCCTCCCAATCTT |
| STAT5a | AGTGCTGACTCCAAACGGG | TCGATGGAGTCCCACGCC |
| STAT5b | TGCAGAAGAAGGCAGAGCA | ATGGGGCAGCGGTCATA |
| STAT6 | GCGTTTCGCCGTCCGTGAG | GCCACGCATTCACTTCTGCC |
| SOX9 | GAGCACTCAGGGCAGTCG | GTACCGCTGTAGGTGGTGAC |
| MUC2 | CCACCTTCGCAACCAGGAAA | TCACGCATACAAAGGAGGAGT |
| DCLK1 | GCTGCGGACATCAGATTGAAC | TTCGGTAGAAGCTGCAGTGG |
| β-actin | CAGCCAGCCATGGATGATGA | CATACCAACCATCACACCCTGA |
